# Supplementary material for: Contribution of the Adenosine 2A Receptor to Behavioral Effects of Tetrahydrocannabinol, Cannabidiol and PECS-101
Source: Molecules. 2021 Sep 2;26(17):5354. doi: 10.3390/molecules26175354 (PMC8434367; doi:10.3390/molecules26175354)
Supplement: Supplementary file 1 [file molecules-26-05354-s001.zip › molecules-1343932-supplementary.pdf]

Supplementary Materials

Contribution of the Adenosine 2A Receptor to Behavioral Effects of Tetrahydrocannabinol, Cannabidiol and PECS-101

Todd M. Stollenwerk, Samantha Pollock and Cecilia J. Hillard*,†

Department of Pharmacology and Toxicology and Neuroscience Research Center, Medical College of Wisconsin, Milwaukee, WI 53226, USA; tstollenwerk@mcw.edu (T.M.S.); smnthpllck@gmail.com (S.P.)
* Correspondence: chillard@mcw.edu
† Mechoulam Awardee in 2011

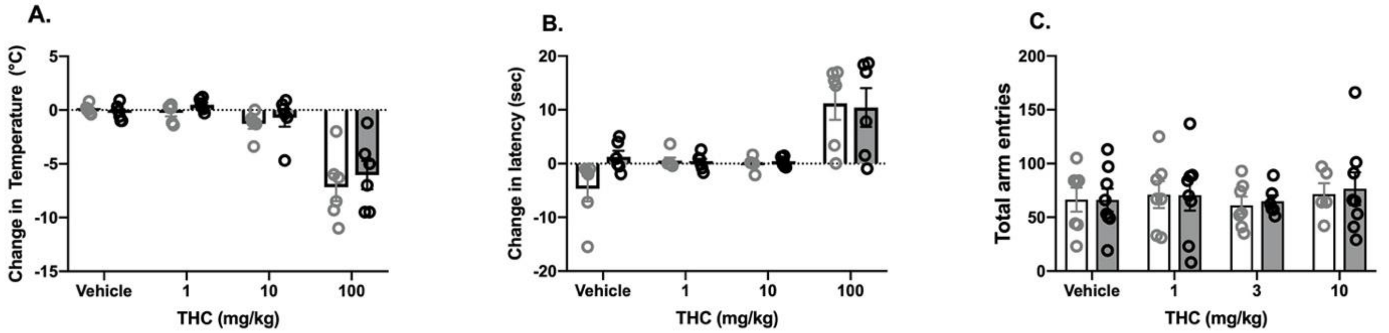

Figure S1. Comparison of behavioral effects of THC in wild type (WT, open bars and gray symbols) and A2AR null (A2AR-KO, closed bars and black symbols) mice. (A.) Effects of THC on rectal temperature. (B). Effects of THC on the latency to move the tail in response to a light; difference between predrug and postdrug latency. (C.) Effects of THC on the total entries into the arms of the EPM. This analysis excluded the mice that were excluded from the OAT/CAT analysis in Figure 1D. A total of 8 mice were removed from this analysis because they entered the open arm and became immobile (1 mouse each from the WT/vehicle, WT/1 mg/kg, WT/3 mg/kg groups; 3 from the WT/10 mg/kg group and 2 from the KO/3 mg/kg group). Bars represent mean and vertical lines represent S.E.M.

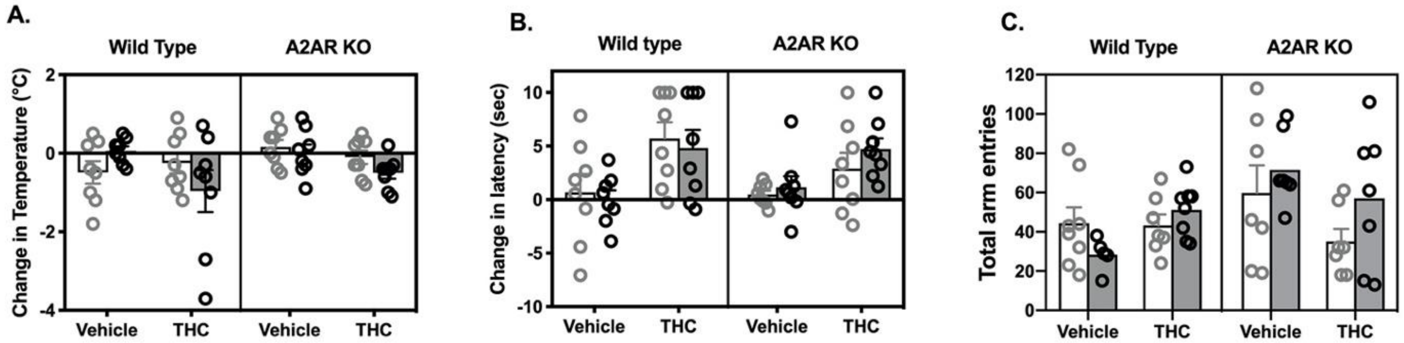

Figure S2. Behavioral effects of THC (5 mg/kg), CBD (5 mg/kg) and their combination in wild type (WT, open bars and gray symbols) and A2AR null (A2AR-KO, closed bars and black symbols) mice. (A.) Drug effects on rectal temperature. (B). Drug effects on the latency to move the tail in response to a light; difference between pre-drug and post-drug latency. (C.) Drug effects on the total entries into the arms of the EPM. This analysis excluded the mice that were excluded from the OAT/CAT analysis in Figure 2D. A total of 8 mice were removed from the analysis; 3 mice became immobile on the open arm (2 in the WT/veh/CBD group and 1 in the KO/THC/CBD group) and 5 additional mice were identified as statistical outliers (1 each in the WT/veh/CBD, WT/veh/THC, KO/veh/veh, KO/veh/THC and KO/veh/CBD groups). Bars represent mean and vertical lines represent S.E.M.

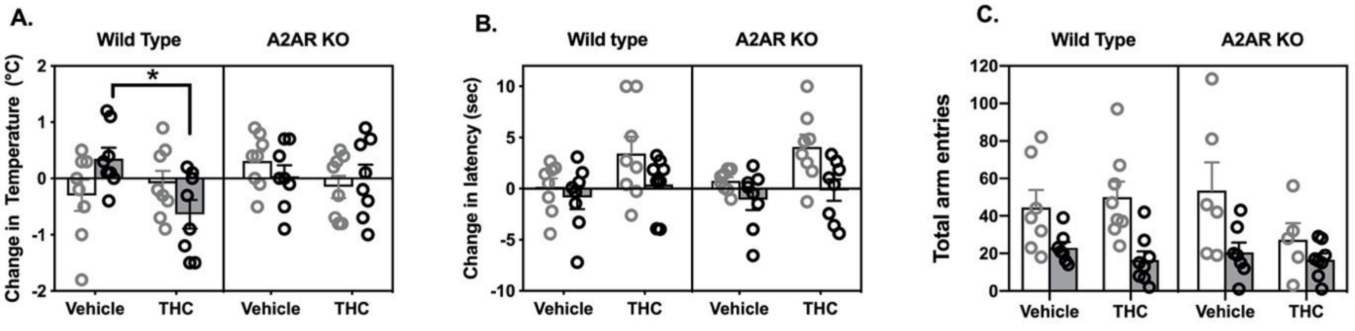

Figure S3. Behavioral effects of THC (5 mg/kg), PECS-101 (5 mg/kg) and their combination in wild type (WT, open bars and gray symbols) and A2AR null (A2AR-KO, closed bars and black symbols) mice. (A.) Drug effects on rectal temperature. (B). Drug effects on the latency to move the tail in response to a light; difference between pre-drug and post-drug latency. (C.) Drug effects on the total entries into the arms of the EPM. This analysis excluded the mice that were excluded from the OAT/CAT analysis in Figure 3D. A total of 8 mice were eliminated from this data set; three mice froze on the open arm of the maze (2 in the KO/veh/PECS-101 group and 1 in the KO/THC/PECS-101 group) and five mice were statistical outliers (one each in the WT/veh/veh, WT/veh/PECS-101 and KO/veh/veh groups; and 2 in the KO/THC/PECS-101 group). * $p<0.05$ Bars represent mean and vertical lines represent S.E.M.

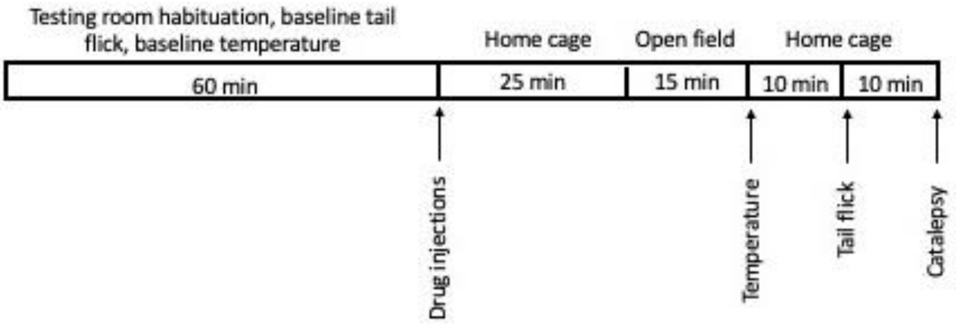

Figure S4. Timeline for the tetrad studies.

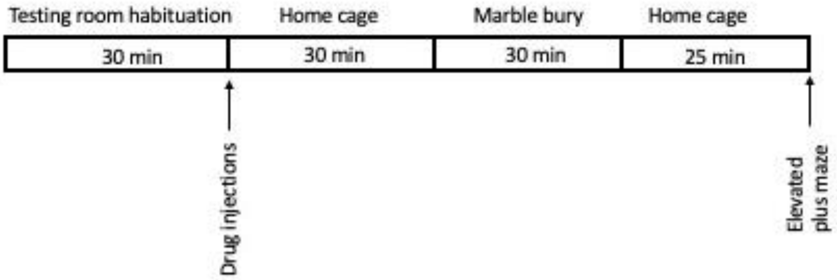

Figure S5. Timeline for the anxiety studies.
